# Association between myalgic encephalomyelitis/chronic fatigue syndrome and irritable bowel syndrome: a systematic review and meta-analysis

**DOI:** 10.3389/fmed.2026.1881784

**Published:** 2026-07-27

**Authors:** Natalie Rajabi, Prakash V. A. K. Ramdass

**Affiliations:** Department of Public Health and Preventive Medicine, St. George’s University School of Medicine, St. George, Grenada

**Keywords:** DGBI, disorder of gut-brain interaction, gut-brain axis, IBS, irritable bowel syndrome, ME/CFS, myalgic encephalomyelitis/chronic fatigue syndrome

## Abstract

**Background:**

Irritable bowel syndrome (IBS) and myalgic encephalomyelitis/chronic fatigue syndrome (ME/CFS) are chronic, debilitating disorders characterized by substantial symptom burden, impaired quality of life, and high healthcare utilization. IBS is a disorder of gut–brain interaction with a global prevalence ranging from 3.8% to 9.2%, while ME/CFS is defined by persistent, exertion-intolerant fatigue and multisystem dysfunction. Growing evidence suggests frequent co-occurrence of these conditions, implicating shared mechanisms such as immune dysregulation, autonomic imbalance, and altered gut–brain signaling. However, the magnitude of their association has not been comprehensively quantified.

**Methods:**

We conducted a systematic review and meta-analysis by searching Embase, PubMed, Scopus, and Google Scholar databases from inception through February 2026. Eligible observational studies reporting associations or prevalence between IBS and ME/CFS were included. Random-effects meta-analyses were performed to pool odds ratios (ORs) and prevalence estimates. Heterogeneity was assessed using I^2^ statistics. We evaluated for publication bias and assessed for risk of bias.

**Results:**

Twenty-two studies were included in the systematic review and 18 in the meta-analysis. Individuals with ME/CFS had significantly higher odds of IBS compared with controls (OR = 7.20; 95% CI: 2.77–18.76; I^2^ = 72.1%). The pooled prevalence of IBS among individuals with ME/CFS was 37% (95% CI: 23–54%), with substantial heterogeneity (I^2^ = 99.5%). Subgroup analysis revealed that diagnostic criteria significantly influenced prevalence (*p* < 0.0001), while meta-regression identified ME/CFS diagnostic criteria as a significant moderator.

**Conclusion:**

IBS and ME/CFS are strongly associated, suggesting shared underlying mechanisms. These findings support integrated screening approaches and coordinated, multidisciplinary management to improve outcomes in affected patients.

**Systematic review registration:**

https://www.crd.york.ac.uk/PROSPERO/view/CRD420251006255.

## 1 Introduction

Irritable bowel syndrome (IBS) is a chronic functional gastrointestinal disorder characterized by recurrent abdominal pain associated with changes in bowel habits, and it is classified as part of the broader group of disorders of gut–brain interaction (DGBI) (1). According to the meta-analysis by Oka et al. (2), IBS represents a significant public health concern, with an estimated global adult prevalence ranging from 3.8% to 9.2%, depending on whether Rome IV or Rome III diagnostic criteria are applied.

Emerging evidence suggests that IBS frequently co-occurs with other multisystem conditions, including myalgic encephalomyelitis/chronic fatigue syndrome (ME/CFS), supporting a shared pathophysiological framework involving immune dysregulation, autonomic dysfunction, and altered gut–brain signaling (3). ME/CFS is a debilitating condition marked by persistent fatigue that worsens with exertion and does not improve with rest (4, 5).

Both IBS and ME/CFS are associated with substantial functional impairment, reduced quality of life, and significant healthcare utilization, reflecting their chronic, relapsing nature and limited disease-modifying treatment options (6, 7). Clarifying the magnitude of the association between these conditions may provide important insights into shared mechanisms, inform clinical screening strategies, and guide future translational research aimed at identifying common therapeutic targets.

Although clinical overlap is recognized, reported prevalence rates of IBS in ME/CFS populations vary widely due to small sample sizes and evolving diagnostic criteria (8, 9). This lack of a definitive, pooled estimate complicates the development of integrated care models and standardized screening. Consequently, a comprehensive synthesis is needed to quantify this association and identify the drivers of reported variability.

Like IBS, the exact cause of ME/CFS remains unclear, and despite extensive research into both conditions, limited evidence exists regarding temporal relationships or causal directionality between them. Therefore, our primary objective of this systematic review and meta-analysis was to evaluate the association between ME/CFS and IBS.

## 2 Materials and methods

### 2.1 Study protocol and registration

This systematic review and meta-analysis were conducted in accordance with the Preferred Reporting Items for Systematic Reviews and Meta-Analyses (PRISMA) guidelines and the PRISMA Protocols (PRISMA-P) statement (10). The study protocol was registered in the PROSPERO database (University of York, United Kingdom; CRD420251006255).

### 2.2 Data sources

A comprehensive literature search was conducted in PubMed, Embase, Scopus, and Google Scholar from database inception to the date of the final search to identify studies evaluating the association between myalgic encephalomyelitis/chronic fatigue syndrome (ME/CFS) and irritable bowel syndrome (IBS). Search strategies were tailored to each database. PubMed: ((“Fatigue Syndrome, Chronic”[Mesh OR “myalgic encephalomyelitis”[Mesh]) AND (“Irritable Bowel Syndrome”[Mesh] OR “irritable colon”[Mesh])); Embase: ((‘chronic fatigue syndrome’/exp. OR ‘myalgic encephalomyelitis’/exp) AND (‘irritable colon’/exp)); Scopus: TITLE-ABS-KEY((“chronic fatigue syndrome” OR “myalgic encephalomyelitis” OR “ME/CFS”) AND (“irritable bowel syndrome” OR IBS OR “irritable colon” OR “disorder of gut-brain interaction”)). For Google Scholar, the following two separate searches were employed: ((“chronic fatigue syndrome”) AND (“irritable bowel syndrome”)) [all words in title]; AND((“myalgic encephalomyelitis”) AND (“irritable bowel syndrome”)) [all words in title]. Results from both Google Scholar searches were merged into a single library and citations were exported for screening.

### 2.3 Study selection and eligibility criteria

Citation records from the four databases were imported into Zotero, and duplicate entries were removed. Two investigators (NR and PR) independently screened titles and abstracts to identify studies examining the relationship between ME/CFS and IBS. Articles meeting initial screening criteria underwent full-text review. Eligible studies included original cohort, case–control, and cross-sectional, whereas clinical investigations, animal studies, case reports, conference proceedings, abstracts, letters, and review articles were excluded. Searches were restricted to English-language publications from database inception through February 28, 2026.

### 2.4 Data extraction

Prevalence and association data were extracted from all eligible studies and entered into Microsoft Excel. Studies were organized into two separate worksheets: (1) ME/CFS associated with IBS; and (2) prevalence of IBS among individuals with ME/CFS. Within each worksheet, data were categorized by study design, country/geographical region, ME/CFS diagnostic criteria, IBS diagnostic criteria, confounders, and sample size. When ME/CFS or IBS criteria were based on history or self-report, they were categorized as “not specified.” Studies that met criteria but did not have relevant quantitative data were included in the systematic review only.

### 2.5 Data synthesis and analysis

RStudio software, version 4.5.0, with the meta and metafor packages, was used to conduct meta-analyses, construct forest plots, and perform sensitivity analyses. Because of substantial heterogeneity across studies—including differences in patient populations, age, sex, and diagnostic criteria—a random-effects model was applied. Statistical heterogeneity was quantified using the I^2^ statistic, derived from Cochran’s Q test, which assesses whether observed variability exceeds that expected by chance alone. I^2^ values ≥ 50% were considered indicative of high heterogeneity. Subgroup meta-analyses were conducted based on IBS diagnostic criteria, ME/CFS diagnostic criteria, study design, geographic region, and study quality as assessed by the Newcastle–Ottawa Scale (NOS) (11). To evaluate the robustness of the pooled estimates and identify potential sources of bias, sensitivity and influence analyses were performed. Leave-one-out analysis was conducted to assess the impact of individual studies on the overall effect size. Influence diagnostics were further explored using the Baujat plot and formal statistical measures, including studentized residuals, Cook’s distance, DFFITS, covariance ratios, and leave-one-out heterogeneity estimates (τ^2^ and QE). Studies with standardized residuals exceeding ±2 were considered potential outliers and were evaluated in sensitivity analyses. To further explore sources of between-study heterogeneity, univariable meta-regression analyses were performed using study-level characteristics. Publication bias was assessed using the funnel plot, Egger’s regression test, and Begg’s rank correlation test (12, 13). A *p* value < 0.05 was considered statistically significant for all analyses.

### 2.6 Assessment of bias

Two investigators (NR and PR) independently assessed study quality and risk of bias using a modified NOS (11). The NOS was initially designed to assess cohort and case–control studies; therefore, a modified version adapted for cross-sectional studies was applied, consistent with approaches used in prior systematic reviews (14). The NOS evaluates studies based on selection of study groups, comparability, and ascertainment of exposure and/or outcome. Overall quality scores were recorded for each study, with higher scores indicating better methodological quality. NOS scores were categorized into low risk of bias (7–9 stars), moderate risk of bias (5–6 stars), and high risk of bias (≤4 stars).

## 3 Results

### 3.1 Included studies

**Figure 1:**
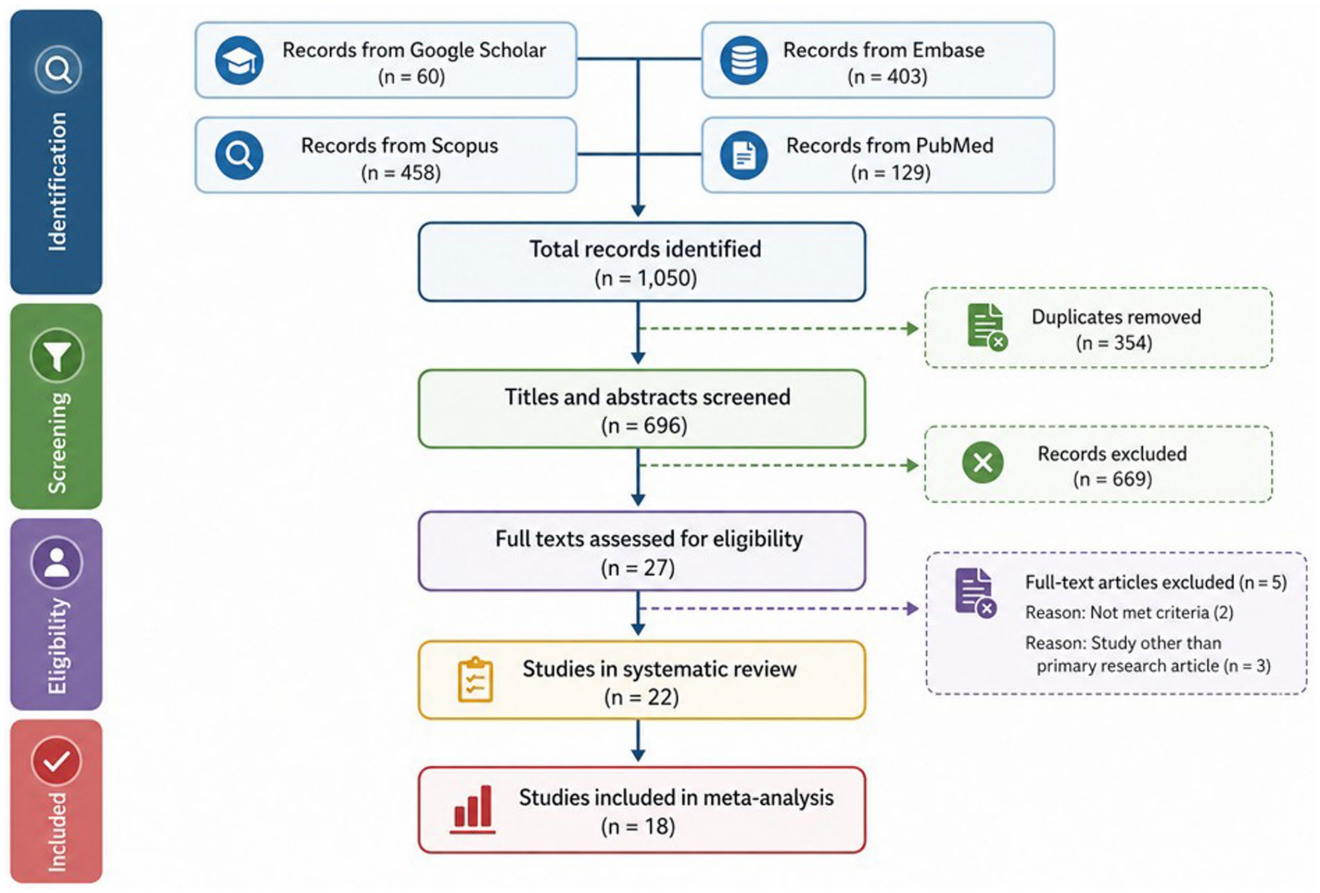
Modified PRISMA flow chart of included studies.

Figure 1 presents the PRISMA flow diagram outlining study selection. A total of 1,050 records were identified, including PubMed (*n* = 129), Embase (*n* = 403), Scopus (*n* = 458), and Google Scholar (*n* = 60). After removal of 354 duplicates, 696 titles and abstracts were screened, of which 669 were excluded. Twenty-seven full-text articles were assessed for eligibility, and five were excluded because they did not meet criteria. Ultimately, 22 studies were included in the systematic review, and 18 studies had sufficient data to be included in the meta-analysis, with 5 studies providing data for the association between ME/CFS and IBS, and 15 studies providing data on the prevalence of IBS among individuals with ME/CFS.

### 3.2 Study characteristics

Table 1 summarizes the characteristics of the included studies (8, 9, 15–34). Most were conducted in Europe and the United States, with one study from Taiwan. Study designs included cross-sectional, case–control, and cohort studies. Diagnostic criteria varied for ME/CFS [e.g., Holmes et al., (35), Fukuda et al., (4), International Consensus Criteria (5), and International Classification of Diseases-9/10 [ICD-9/10] (36, 37)] and IBS (e.g., Manning (38), Rome II/III/IV (1, 39, 40), ICD-9/10 (36, 37)). Many studies did not specify what diagnostic criteria were used for ME/CFS and IBS. Sample sizes ranged from 25 participants to population-based cohorts exceeding one million individuals. The studies adjusted for several potential confounders, most frequently including demographic characteristics such as age and sex, body mass index (BMI), pre-existing medical and psychiatric comorbidities, and current medication use. Overall, substantial methodological and clinical heterogeneity was observed, with most studies reported a predominance of female participants.

**Table 1:** Characteristics of included studies.

Study	Country	Study design	ME/CFS criteria	IBS criteria	Confounders	Sample size
Meta-analysis						
Gomborone et al. (15)	United Kingdom	Cross-sectional	Not specified	Manning	GI history	1,129
Aaron et al. (9)	United States	Case–Control	Holmes	Manning	Age, sex	25
Van Oudenhove et al. (16)	Belgium	Cross-sectional	Not specified	Rome II	Function, abuse, psychiatric	141
Dansie et al. (17)	United States	Cross-sectional	Fukuda	Rome II	Age, gender, twins	123
Maes et al. (18)	Belgium	Case–Control	Fukuda	Rome II	Age, gender	94
Janssens et al. (19)	Netherlands	Prospective cohort	History	History	Age, sex	1,166
Nagy-Szaka et al. (20)	United States	Case–Control	Fukuda	Self-reported	Demographics, BMI	50
Chu et al. (21)	United States	Cross-sectional	Fukuda	Not specified	Infection, stress, toxins	150
Kenyon et al. (8)	United Kingdom	Retrospective Cohort	ICC	Not specified	Comorbidities, medications	42
Berstad et al. (22)	Norway	Cross-sectional	ICC	Rome III	Age, sex, BMI	1,100
Ghali et al. (23)	France	Retrospective Cohort	ICC	Not specified	Onset, delay, comorbidities	168
Kendler et al. (24)	Sweden	Cross-sectional	ICD-10	ICD-10	Socio-demographics, medications	31,578
Monden et al. (25)	Netherlands	Prospective cohort	History	History	Demographics, BMI, antibiotics	296
Guo et al. (26)	United States	Case–control	Fukuda	Self-reported	Sex, year, cohabitation	106
Tarar et al. (27)	United States	Retrospective Cohort	ICD-10	Rome IV	Demographics, comorbidities	1,256,325
Fall et al. (28)	United States	Cross-sectional	Fukuda	Not specified	Age, sex	595
Huang et al. (29)	United Kingdom	Cross-sectional	Self-reported	Self-reported	Age, sex, medications	1,194
van der Meulen et al. (30)	Netherlands	Cross-sectional	Fukuda	Rome IV	Chronicity	2,804
Systematic review only						
Hamilton et al. (31)	United Kingdom	Case–Control	GPRD codes	GPRD codes	Age, gender	8,776
Chang et al. (32)	Taiwan	Retrospective Cohort	ICD-9/10	ICD-9/10	Age, sex, comorbidities	33,372
Saini et al. (33)	Netherlands	Retrospective Cohort	Fukuda	Rome III	Age, sex	72,919
Thomas et al. (34)	Netherlands	Prospective cohort	Fukuda	Rome III	Age, sex	88,457

### 3.3 Systematic review

Across 22 studies, there is consistent evidence of substantial diagnostic overlap between ME/CFS and IBS, suggesting shared underlying pathophysiological mechanisms. IBS prevalence among ME/CFS patients ranged from 38% to 92%, markedly exceeding rates observed in the general population. Conversely, individuals with IBS also demonstrate increased rates of ME/CFS in observational and registry-based studies (27, 31), with co-occurrence estimates substantially exceeding those expected by chance. However, these findings reflect association rather than a clearly established directional risk relationship. This relationship is characterized by shared symptom profiles, particularly abdominal pain, while fatigue, cognitive impairment, and unrefreshing sleep act as key “bridge symptoms” linking both conditions. Clinically, IBS symptoms often precede ME/CFS, although distinct proximal triggers are observed, including gastroenteritis for IBS and viral infections for ME/CFS.

Biological evidence further supports this overlap, with patients exhibiting increased intestinal permeability, systemic inflammation, and distinct metabolomic alterations, including elevated ceramides and disrupted short-chain fatty acid metabolism. Psychological factors such as somatization also contribute to both conditions.

Collectively, these findings support the concept that ME/CFS–IBS comorbidity represents a more severe and systemic manifestation of dysfunction within the gut–brain axis.

### 3.4 Meta-analysis: association of ME/CFS and IBS

**Figure 2:**
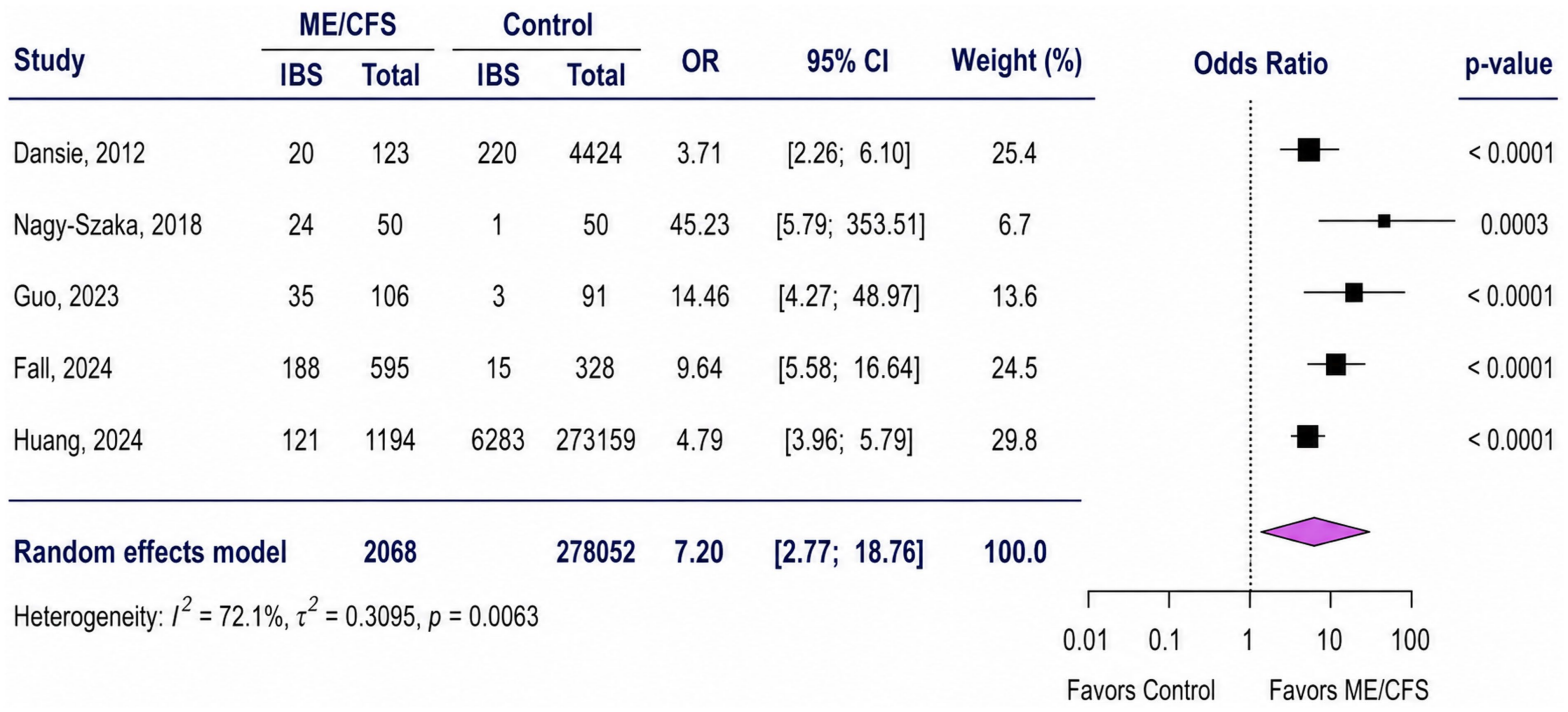
Odds of ME/CFS and IBS.

Figure 2 shows that the pooled random-effects analysis demonstrated a strong association between ME/CFS and IBS, with individuals with ME/CFS having more than sevenfold higher odds of IBS compared with controls (OR = 7.20; 95% CI: 2.77–18.76, *p* < 0.001). All five included studies reported significantly elevated odds, with individual effect estimates ranging from 3.71 to 45.23. Larger, population-based studies contributed the greatest statistical weight and provided more precise estimates, whereas smaller studies yielded wider confidence intervals and more extreme values. Although heterogeneity was substantial (I^2^ = 72.1%), likely reflecting variation in study design, populations, and diagnostic criteria, the direction of effect was consistent across studies, supporting a robust and clinically meaningful association between ME/CFS and IBS.

### 3.5 Meta-analysis: prevalence of IBS in ME/CFS according to IBS criteria

**Figure 3:**
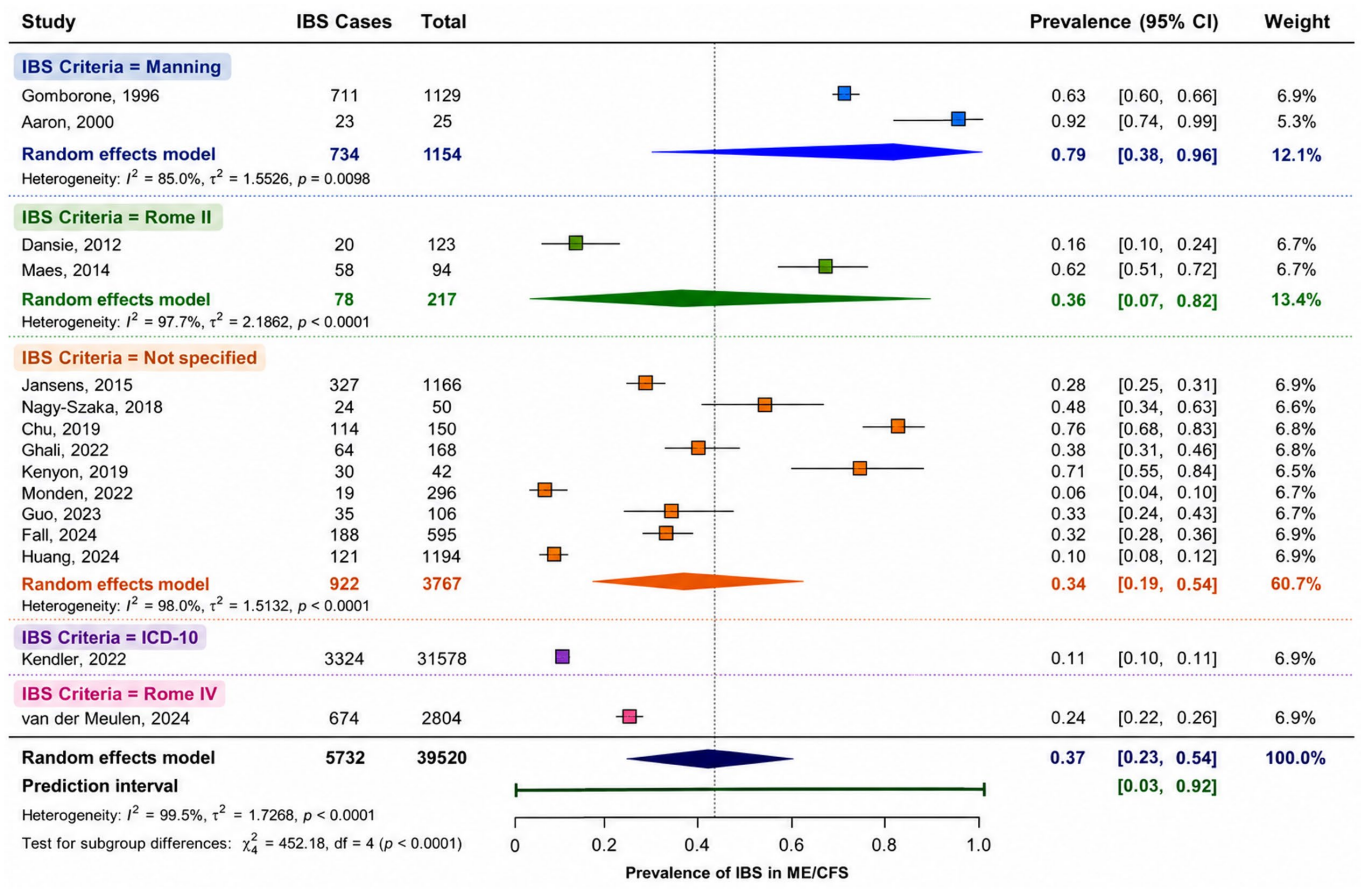
Prevalence of IBS in patients with ME/CFS according to IBS criteria.

Figure 3 presents the subgroup meta-analysis of IBS prevalence in individuals with ME/CFS stratified by IBS diagnostic criteria. The overall pooled prevalence was 0.37 (95% CI: 0.23–0.54); however, heterogeneity was extreme (I^2^ = 99.5%), indicating substantial variability in prevalence estimates across studies. The prediction interval ranged from 0.03 to 0.92, suggesting that the prevalence of IBS in future ME/CFS populations may vary considerably depending on the study setting, population characteristics, and diagnostic definitions employed. Prevalence estimates varied across diagnostic subgroups, with higher pooled estimates observed for Manning criteria (38) (0.79, 95% CI: 0.38–0.96) compared with Rome II (39) (0.36, 95% CI: 0.07–0.82) and studies with unspecified criteria (0.34, 95% CI: 0.19–0.54). Single-study subgroups showed lower prevalence using Rome IV (1) (0.24) and ICD-10 criteria (36) (0.11). There was a significant difference between subgroups (*p* < 0.0001), indicating that IBS prevalence estimates are influenced by the diagnostic criteria used.

### 3.6 Meta-analysis: prevalence of IBS in ME/CFS according to ME/CFS criteria

**Figure 4:**
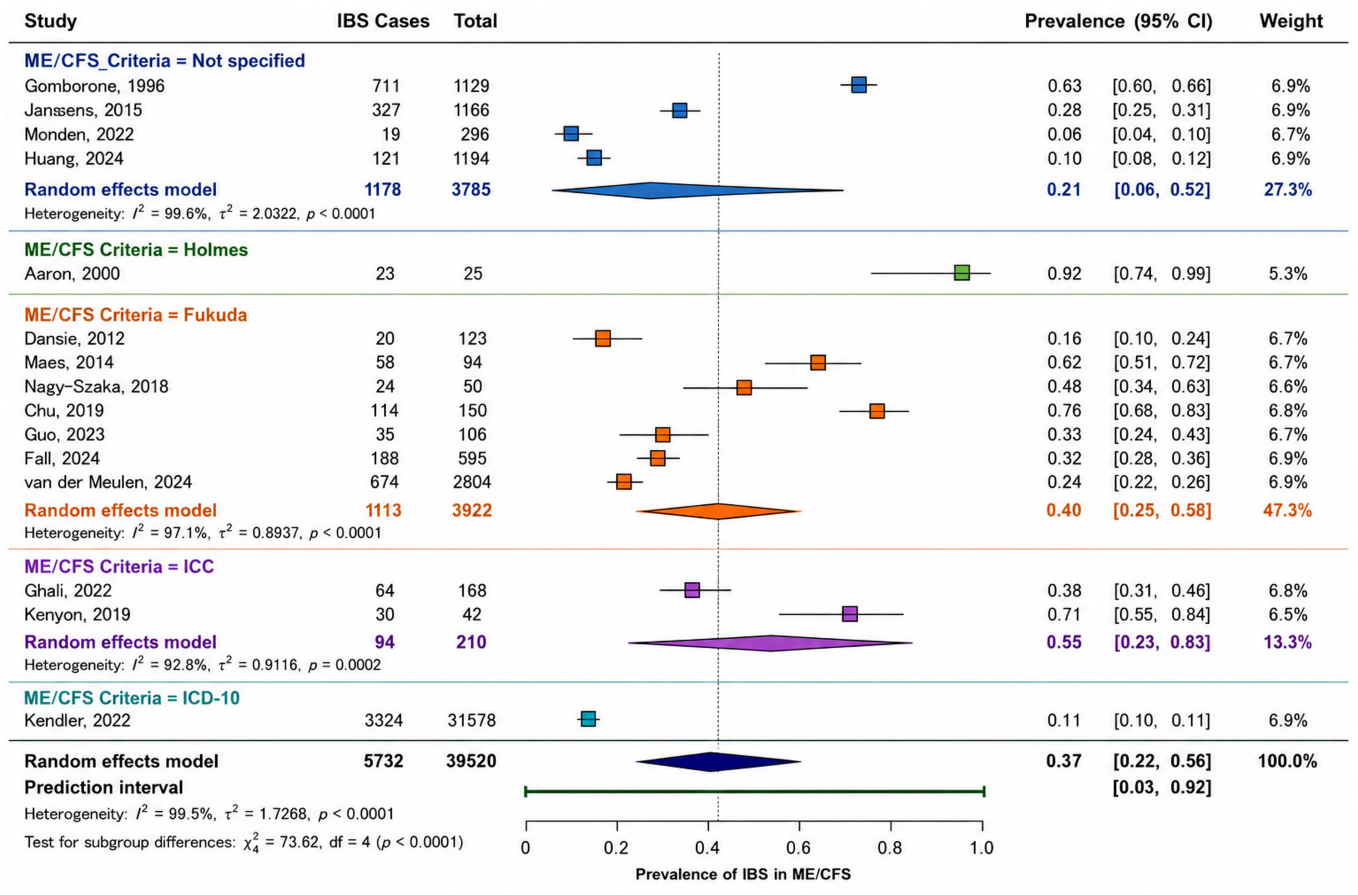
Prevalence of IBS in patients with ME/CFS according to ME/CFS criteria.

As shown in Figure 4, subgroup analysis by ME/CFS diagnostic criteria demonstrated variability in the pooled prevalence of IBS. Higher prevalence estimates were observed in studies using ICC criteria (0.55, 95% CI: 0.23–0.83) and Fukuda criteria (4) (0.40, 95% CI: 0.25–0.58), whereas lower estimates were seen in studies with unspecified criteria (0.21, 95% CI: 0.06–0.52) and ICD-10 criteria (36) (0.11). A single study using Holmes’ criteria (35) reported a notably high prevalence (0.92). There was a significant difference between subgroups (*p* < 0.0001), indicating that IBS prevalence in ME/CFS varies according to the diagnostic criteria applied.

### 3.7 Subgroup analyses

Subgroup analyses by study design, geographic region, and risk of bias are presented in Supplementary Figures S1–S3. Prevalence estimates varied by study design (highest in case–control [0.60] and lowest in prospective cohort [0.14]), geographic region (USA: 0.49; Europe: 0.30), and risk of bias (high-risk: 0.54; low/moderate-risk: 0.33). However, none of these differences reached statistical significance (*p* = 0.1105, *p* = 0.2365, and *p* = 0.4972, respectively), indicating that study design, region, and study quality did not significantly modify the pooled prevalence estimates. Heterogeneity remained very high across all analyses (I^2^ > 98%), suggesting that the examined characteristics explained only a small fraction of the observed variability.

### 3.8 Sensitivity analysis: association of ME/CFS and IBS

**Figure 5:**
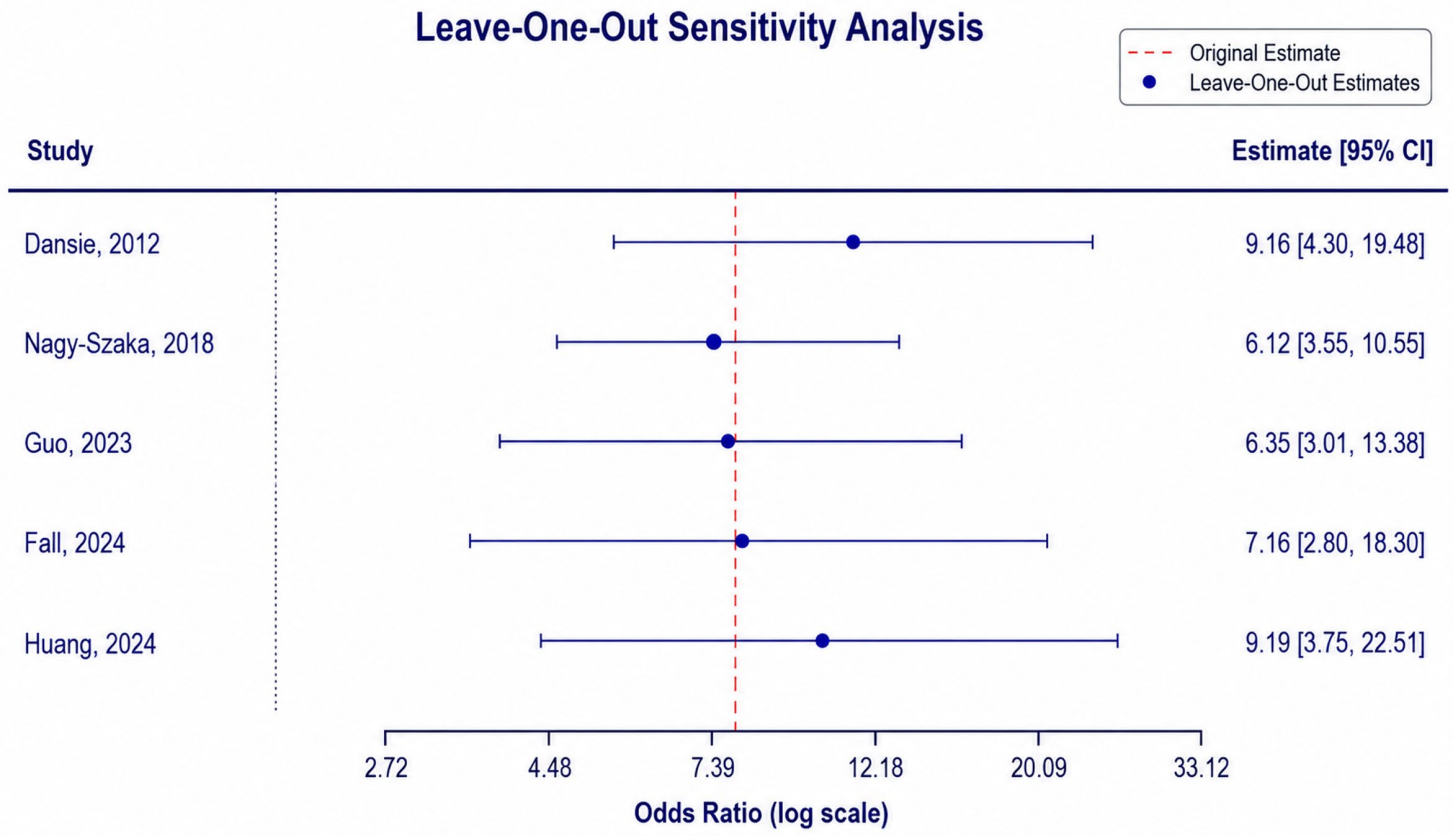
Leave-one-out sensitivity analysis for Odds of ME/CFS and IBS.

The leave-one-out analysis, shown in Figure 5, demonstrated that the association between ME/CFS and IBS remained statistically significant regardless of which study was excluded. Pooled odds ratios ranged from 6.1 to 9.2, indicating robust findings.

### 3.9 Sensitivity analysis: prevalence of IBS in ME/CFS

**Figure 6:**
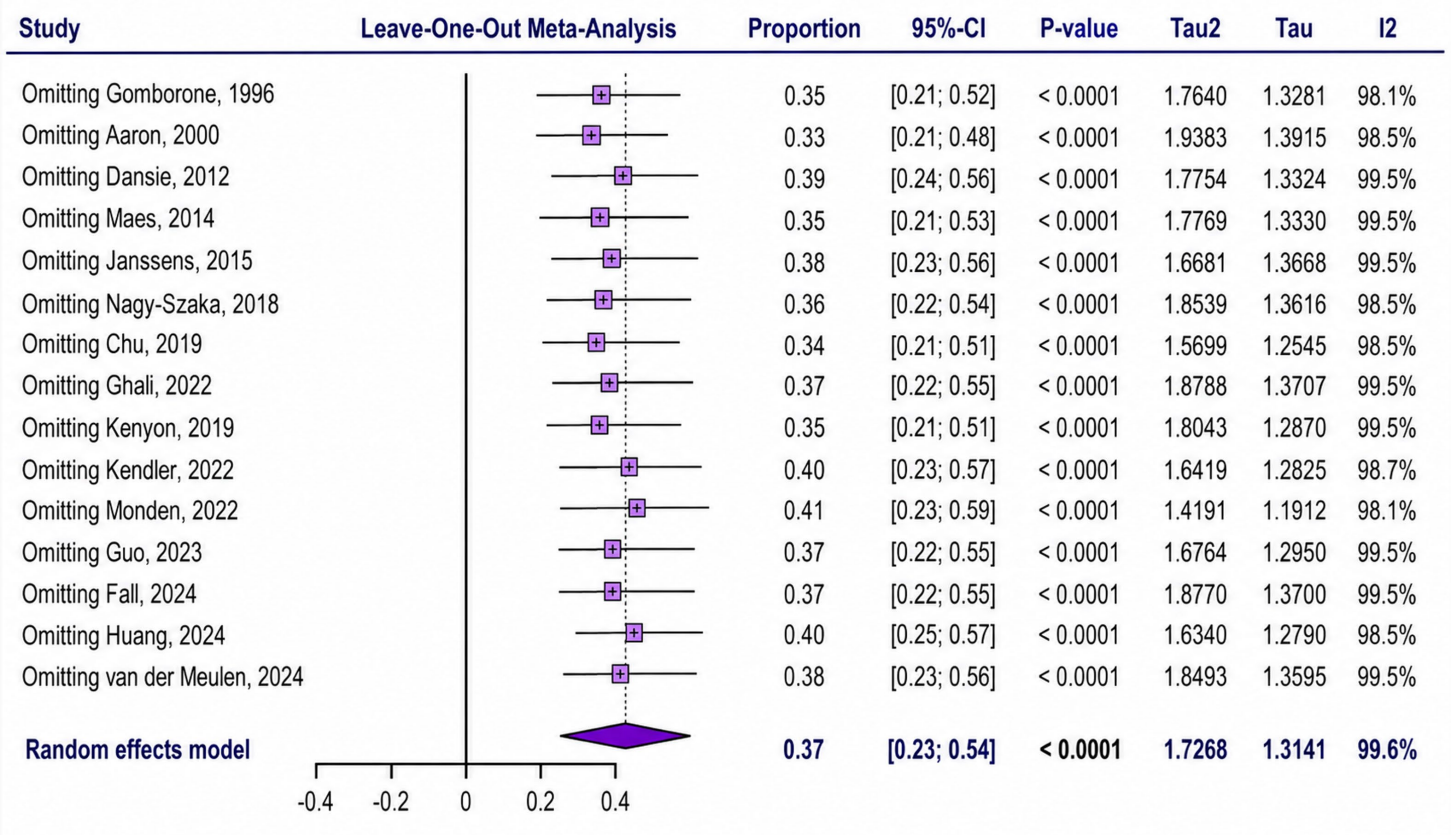
Leave-one-out sensitivity analysis for prevalence of IBS in patients with ME/CFS.

The leave-one-out sensitivity analysis in Figure 6 shows that the pooled prevalence estimate (0.37, 95% CI 0.23–0.54) remained stable when any single study was removed, with recalculated estimates ranging from 0.33 to 0.40. Heterogeneity remained extreme (I^2^ > 99%).

### 3.10 Influence diagnostics and Baujat plot

**Figure 7:**
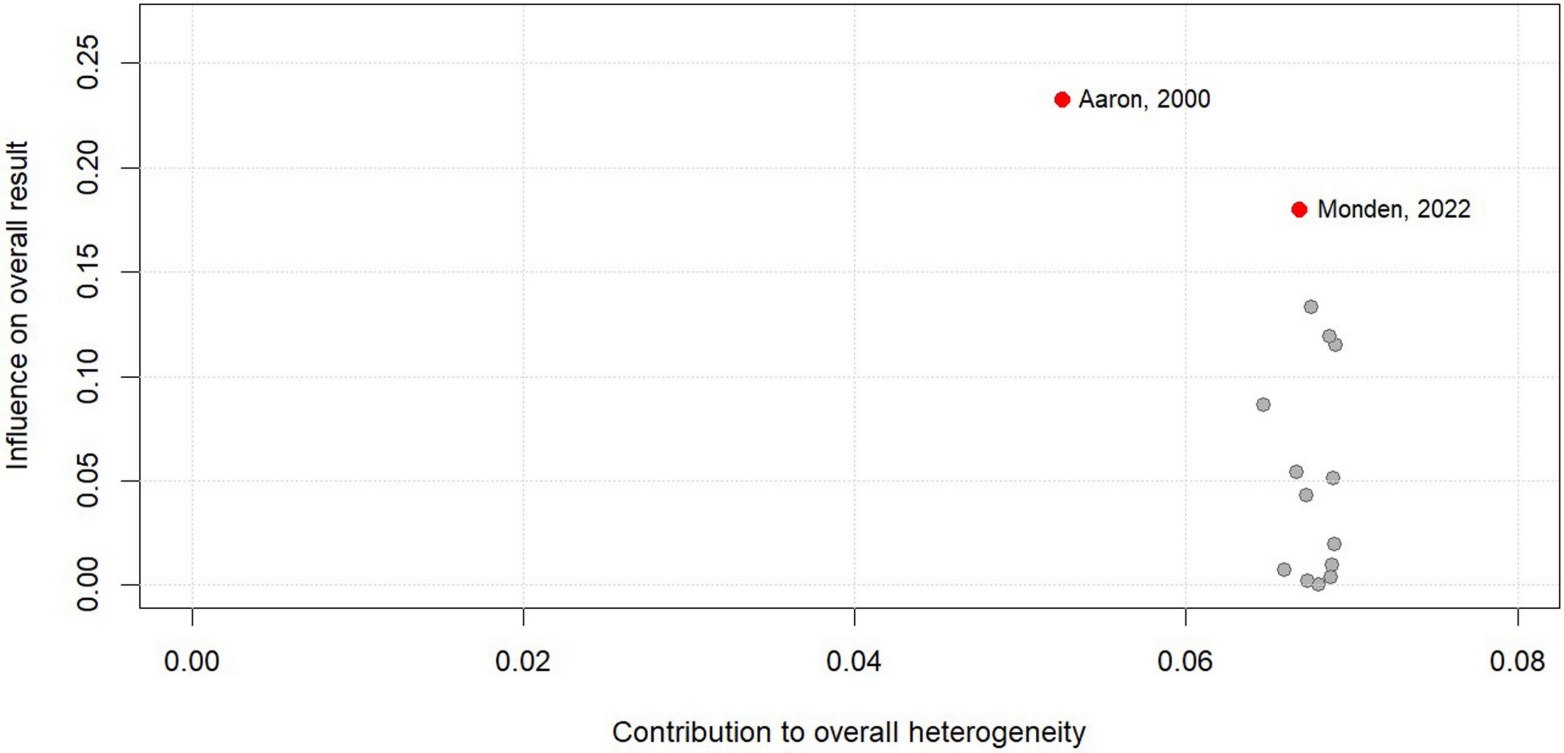
Baujat plot of overall heterogeneity and influence diagnostics.

Influence diagnostics identified a small number of studies exerting disproportionate effects on the pooled estimate and heterogeneity, as demonstrated in the Baujat plot in Figure 7, with Aaron et al. (9) and Monden et al. (25) showing the greatest influence. The majority of studies clustered with minimal contribution to heterogeneity, indicating that the overall findings were not driven by a single dominant study.

### 3.11 Meta-regression analysis

Exploratory meta-regression analyses demonstrated that heterogeneity remained substantial across all models (I^2^ > 98%). Among the examined moderators, ME/CFS diagnostic criteria significantly influenced IBS prevalence (*p* = 0.044), accounting for approximately 28% of between-study heterogeneity, with studies using the Holmes criteria (35) reporting higher prevalence estimates. In contrast, IBS diagnostic criteria (1, 35, 39, 40) were not a significant moderator (*p* = 0.211), explaining only a small proportion of variability (8%), although a trend toward higher prevalence with Manning criteria (38) was observed. Study design showed a modest effect, with prospective cohort studies reporting lower prevalence (*p* = 0.041), but the overall moderator effect was not statistically significant. Geographic region did not contribute meaningfully to heterogeneity. Overall, these findings indicate that while methodological differences—particularly ME/CFS diagnostic criteria—partially explain variability, a large proportion of heterogeneity remains unexplained.

### 3.12 Publication bias

**Figure 8:**
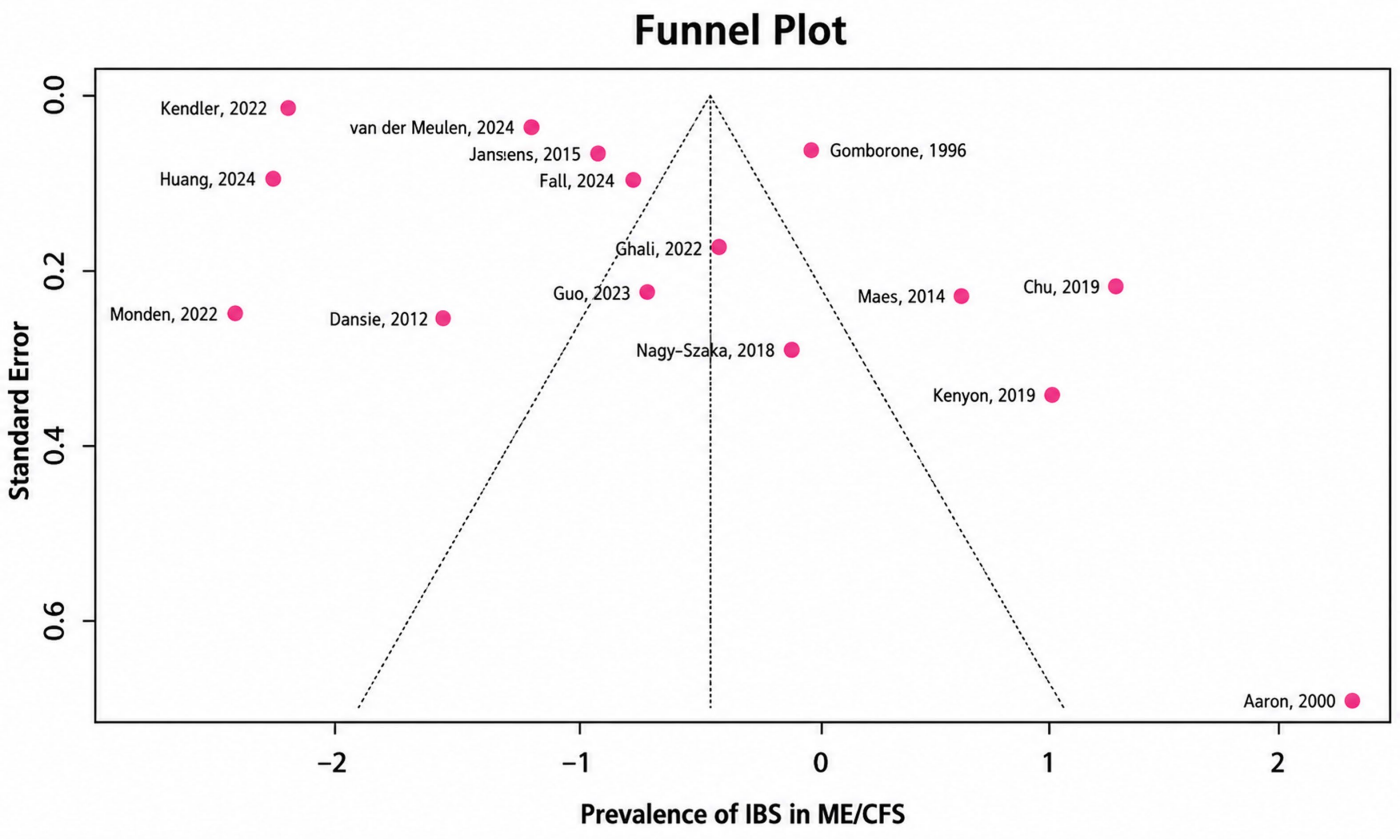
Funnel plot for publication bias.

Visual inspection of the funnel plot in Figure 8 appears asymmetrical, with smaller studies reporting higher prevalence estimates. Egger’s test (12) indicated significant asymmetry (*p* = 0.0197), whereas Begg’s test (13) was nonsignificant (*p* = 0.299). These findings suggest small-study effects and potential publication bias, and thus interpretation of the findings should be cautious.

### 3.13 Assessment of bias

**Figure 9:**
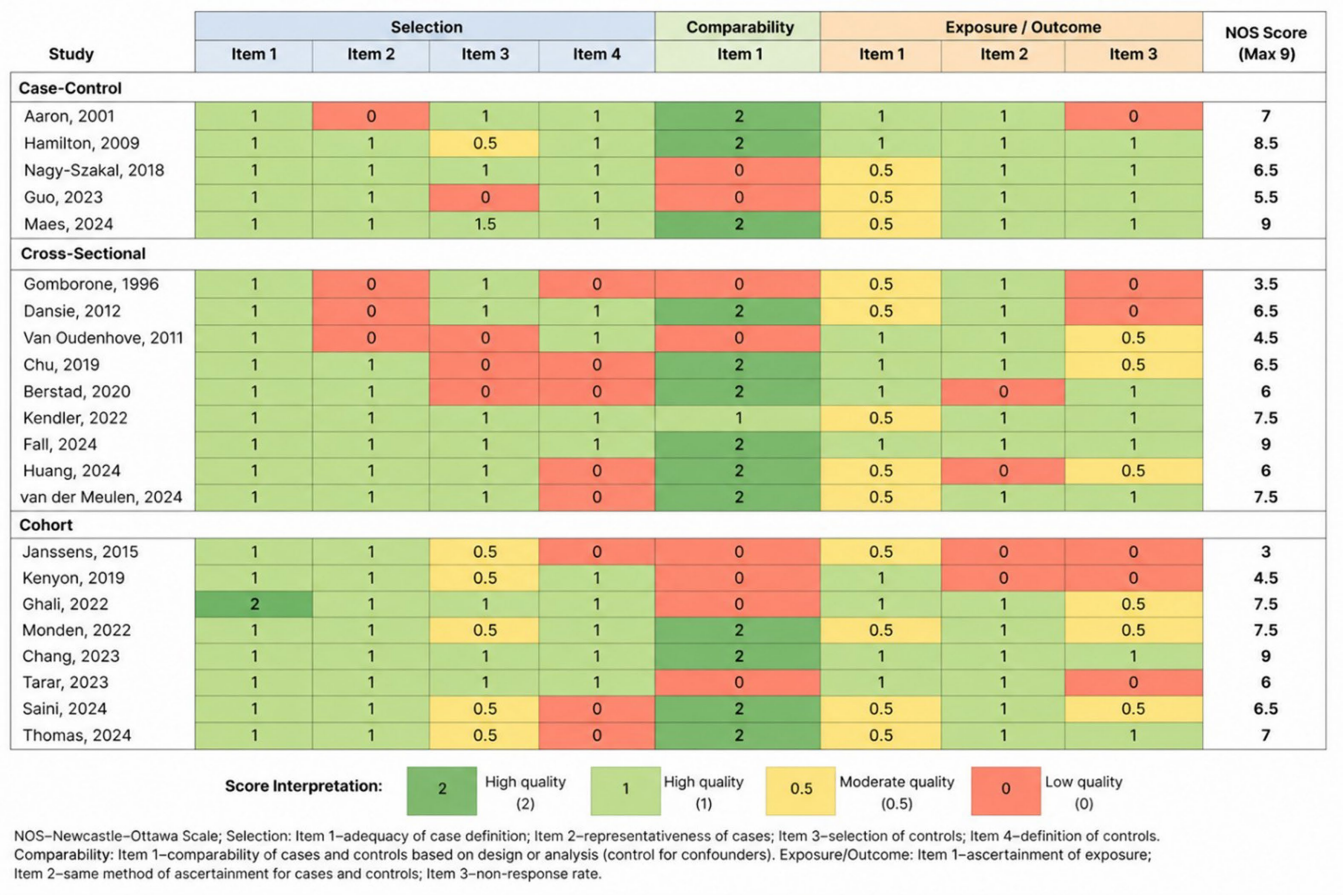
Risk of bias assessment according to Newcastle-Ottawa Scale.

Figure 9 presents the methodological quality of studies using the NOS (11, 14) across three domains. Overall, study quality ranged from low to high (NOS scores: 3.0–9.0). Among case–control studies, scores ranged from 5.5 to 9.0. Cross-sectional studies demonstrated wider variability (3.5–9.0). Cohort studies generally exhibited moderate to high quality (3.0–9.0). Most studies performed well in selection and exposure/outcome domains, whereas comparability was more inconsistent, indicating potential residual confounding in several investigations.

## 4 Discussion

This systematic review and meta-analysis provides the most comprehensive quantitative synthesis to date on the association between ME/CFS and IBS. The key finding—a more than sevenfold higher odds of IBS among individuals with ME/CFS, alongside a pooled IBS prevalence of 37%—indicates that these conditions co-occur far more frequently than expected by chance. From a neurogastroenterology perspective, this degree of comorbidity suggests shared pathophysiological mechanisms within the gut–brain axis rather than simple diagnostic overlap. The predominance of female participants across studies (22, 27, 28) aligns with the higher prevalence of both IBS (41) and ME/CFS (42) in women and suggests potential sex-specific susceptibility, possibly driven by differences in immune response, sex hormones, and gut–brain axis regulation, alongside psychosocial factors.

The association identified in this meta-analysis, particularly the higher prevalence of IBS in cohorts defined using criteria that emphasize autonomic dysfunction (ICC) (5), points toward autonomic imbalance as an important shared mechanism. One possible explanation is a “dysbiotic march,” in which impaired autonomic regulation contributes to increased intestinal permeability and low-grade systemic inflammation, thereby amplifying neuroimmune responses linked to fatigue and cognitive dysfunction (22). While this model remains hypothetical, it offers a useful framework for interpreting the observed overlap.

Taken together, these findings suggest that IBS and ME/CFS may share overlapping biological pathways and clinical features within the gut–brain axis (43). However, the current evidence is insufficient to conclude that they represent a single disease spectrum. Rather, the available data support the concept of distinct disorders with substantial clinical and pathophysiological overlap, potentially affecting specific patient subgroups to varying degrees. In IBS, visceral hypersensitivity and altered central pain processing are well recognized (44, 45) whereas ME/CFS is characterized by central sensitization, autonomic dysregulation, and impaired descending pain modulation (46, 47). Against this background, it is plausible that a subset of patients with ME/CFS exhibits a neuroenteric phenotype, in which dysfunction extends beyond the central nervous system to involve enteric neural circuits and gut–brain signalling pathways (48). This may help explain gastrointestinal symptoms such as abdominal pain and altered bowel habits in these patients. Conversely, IBS patients with severe, persistent fatigue may represent an underrecognized subgroup with overlapping ME/CFS features (27, 43). The relatively high prevalence of IBS in ME/CFS—affecting more than one in three patients—supports the case for routine clinical screening in both directions, although this is not yet standard practice.

While these mechanisms provide biologically plausible explanations for the observed overlap between ME/CFS and IBS, they should be interpreted cautiously. The studies included in this meta-analysis primarily evaluated epidemiological associations and did not directly assess gut microbiota composition, microbial metabolites, autonomic function, immune activation, or other mechanistic biomarkers. Consequently, the present findings support an association between ME/CFS and IBS but do not establish causality, temporal relationships, or a common biological pathway. The proposed mechanisms should therefore be regarded as hypotheses that may explain the observed comorbidity and warrant further investigation in longitudinal and mechanistic studies.

Several lines of evidence converge on the gut–brain axis as a shared vulnerability. The gut microbiome, in particular, has emerged as a central component of dysregulation in both disorders (26, 49, 50). Metagenomic studies consistently demonstrate reduced abundance of butyrate-producing taxa, such as *Faecalibacterium prausnitzii* and *Eubacterium rectale*, along with decreased short-chain fatty acid production in ME/CFS (26), with emerging evidence also suggesting alterations in fungal communities, including taxa such as *Candida* species (51). Importantly, lower abundance of butyrate-producing organisms correlates with greater fatigue severity, supporting a mechanistic link between gut dysbiosis and symptom burden; these abnormalities may be more pronounced in individuals with coexisting gastrointestinal symptoms, including IBS (52). Similarly, IBS is characterized by reduced microbial diversity and alterations in community structure that correlate with symptom severity (49, 53). Butyrate deficiency is especially relevant, given its role in maintaining intestinal barrier integrity, modulating immune responses, and influencing enteric neuronal excitability (54). Whether these changes directly contribute to visceral hypersensitivity or altered gastrointestinal motility in patients with overlapping ME/CFS and IBS remains unclear and warrants further investigation.

Immune dysregulation provides another plausible link between the two conditions. Both IBS and ME/CFS have been associated with low-grade systemic inflammation, increased intestinal permeability, and altered innate immune responses (55). Patients with comorbid disease appear to exhibit greater oxidative and nitrosative stress, as well as more pronounced immune activation, compared with those with either condition alone (56, 57). From a clinical perspective, these observations raise the possibility that immunomodulatory or anti-inflammatory approaches—already explored in subsets of IBS—may have broader benefits in patients with overlapping syndromes (58, 59). However, direct evidence from clinical trials is currently lacking.

Autonomic dysfunction, a hallmark of ME/CFS, may also contribute to gastrointestinal symptoms through multiple interconnected mechanisms involving parasympathetic withdrawal and sympathetic overactivity (60). Reduced vagal (parasympathetic) tone can impair gastric accommodation and delay gastric emptying, while dysregulated autonomic signalling may disrupt coordinated enteric neural activity, leading to altered colonic transit and bowel dysmotility (61). Recent evidence demonstrates that patients with ME/CFS exhibit a substantial burden of dysautonomia across cardiovascular and neurovegetative domains, supporting widespread autonomic imbalance (46). More broadly, autonomic dysfunction has been directly linked to abnormalities in gastrointestinal motility, including delayed gastric emptying and impaired digestive responses, which can manifest as nausea, bloating, and early satiety (62). Mechanistically, disrupted bidirectional signalling along the gut–brain axis may alter enteric neuronal excitability and smooth muscle function, thereby contributing to both upper and lower gastrointestinal symptoms (63). Despite this biologically plausible framework, none of the studies included in this review directly assessed objective measures of gastrointestinal motility or autonomic function in patients with coexisting ME/CFS and IBS, highlighting a critical gap in the literature and an important area for future investigation.

This absence of physiological data is particularly noteworthy because genetic and familial studies (64, 65)—which do not require such measurements—have independently converged on a compelling case for shared biological vulnerability. Genetic and familial studies further support a shared vulnerability between IBS and ME/CFS (66). Twin and family studies demonstrate moderate heritability for both conditions and indicate overlapping genetic risk across immune, pain, psychiatric, and internalizing pathways (24). Population-based registry analyses similarly show that clustering of IBS, ME/CFS, and related functional somatic syndromes occurs at rates substantially higher than expected by chance (31). Collectively, these findings suggest that IBS and ME/CFS share nonspecific but overlapping heritable predispositions rather than a single disease-specific genetic mechanism.

The substantial heterogeneity observed across studies (I^2^ = 99.5% for prevalence estimates) requires careful interpretation. Subgroup analyses indicated that differences in diagnostic criteria for both IBS and ME/CFS had a meaningful impact on prevalence estimates, whereas study design, geographic region, and risk of bias did not. As expected, broader definition, such as the Manning criteria (38) for IBS, and Holmes et al. (35) or Fukuda et al. (4) criteria for ME/CFS, were associated with higher prevalence estimates compared with more restrictive definitions, including Rome IV (1) and the International Consensus Criteria (5), for IBS and Me/CFS, respectively. This pattern underscores the importance of diagnostic frameworks in shaping epidemiological findings. These diagnostic frameworks differ considerably in their sensitivity and specificity, and older or broader definitions may capture more heterogeneous patient populations than contemporary criteria. Consistent with this, our subgroup analyses demonstrated significant differences in IBS prevalence according to both IBS and ME/CFS diagnostic criteria, while meta-regression identified ME/CFS diagnostic criteria as a significant moderator of prevalence estimates. Consequently, part of the observed association between IBS and ME/CFS may reflect differences in case ascertainment rather than true biological variation. Future studies should prioritize standardized diagnostic criteria to improve comparability across populations and generate more precise estimates of comorbidity. For both research and clinical practice, the use of standardized and contemporary criteria is essential to ensure comparability and to avoid overestimation of comorbidity.

Several limitations should be emphasized. The most important limitation of this meta-analysis is the extreme between-study heterogeneity observed in the prevalence analysis (I^2^ = 99.5%). Such heterogeneity suggests that the included studies may not be sampling the same underlying population and likely reflect important differences in study design, patient characteristics, healthcare settings, diagnostic criteria, and methods of case ascertainment. Consequently, the pooled prevalence estimate of 37% should be interpreted as a descriptive summary of the available literature rather than a precise estimate of the true prevalence of IBS among individuals with ME/CFS.

Furthermore, most included studies were cross-sectional, limiting any inference regarding temporal or causal relationships. Although the high degree of comorbidity observed in this review supports the existence of shared mechanisms, the predominantly observational and cross-sectional nature of the evidence precludes conclusions regarding causality or whether IBS and ME/CFS represent manifestations of a common disease process. Moreover, the two prospective cohort studies (19, 25) that examined the risk of IBS in patients with ME/CFS did not clearly specify the diagnostic criteria used for either condition. In contrast, the only prospective cohort study employing clearly defined diagnostic criteria for both ME/CFS and IBS was reported by Thomas et al. (34). While this study applied the Fukuda criteria (4) for ME/CFS and the Rome III criteria (40) for IBS, it evaluated the odds of association without establishing a clear risk factor–outcome relationship or specifying the direction of the association. A further limitation is that none of the included studies applied the more recent Institute of Medicine (National Academy of Medicine) diagnostic criteria (67) for ME/CFS, potentially limiting the clinical relevance and comparability of findings to current diagnostic standards. It remains unclear whether IBS precedes, follows, or develops concurrently with ME/CFS in most cases, and whether treating one condition influences the course of the other. In addition, none of the studies incorporated objective measures of gastrointestinal function, such as motility, transit time, or visceral sensitivity.

Finally, publication bias was also evident, with smaller studies tending to report higher prevalence estimates. The significant Egger’s test further suggests the presence of small-study effects, whereby smaller studies reported disproportionately higher prevalence estimates than larger studies. Consequently, the pooled prevalence of 37% may be somewhat inflated and should be interpreted cautiously. Larger, population-based studies generally reported lower prevalence estimates, suggesting that the true prevalence of IBS among individuals with ME/CFS may be lower than the pooled estimate observed in this meta-analysis.

Despite these limitations, the findings have clear implications for clinical practice. Given that a substantial proportion of individuals with ME/CFS meet criteria for IBS, routine screening using validated tools, such as the Rome IV questionnaire (1), may be warranted. Similarly, IBS patients presenting with profound fatigue, post-exertional malaise, or unrefreshing sleep should be evaluated for possible ME/CFS with the most recent criteria (67). An integrated, contemporary, multidisciplinary approach, combining dietary strategies, behavioural therapies, neuromodulators, and activity management, may be more effective than isolated treatment strategies, although prospective studies are needed to confirm this. Future longitudinal studies incorporating standardized diagnostic criteria, biomarkers, and objective physiological assessments are needed to better characterize the nature of this overlap.

## 5 Conclusion

This meta-analysis demonstrates a strong and clinically meaningful association between IBS and ME/CFS, supporting the presence of shared neuroimmune and gut–brain mechanisms. These findings reinforce the need for integrated clinical approaches and highlight important directions for future research, particularly studies incorporating objective physiological measures to better define the overlap between these conditions.

## Data Availability

The raw data supporting the conclusions of this article will be made available by the authors, without undue reservation.

## Supplementary material

SUPPLEMENTARY FIGURE S1 Prevalence of IBS in patients with ME/CFS according to study design.

SUPPLEMENTARY FIGURE S2 Prevalence of IBS in patients with ME/CFS according to study region.

SUPPLEMENTARY FIGURE S3 Prevalence of IBS in patients with ME/CFS according to risk of bias assessment.
